# Novel Hominid-Specific IAPP Isoforms: Potential Biomarkers of Early Alzheimer’s Disease and Inhibitors of Amyloid Formation

**DOI:** 10.3390/biom13010167

**Published:** 2023-01-13

**Authors:** Qing-Rong Liu, Min Zhu, Qinghua Chen, Maja Mustapic, Dimitrios Kapogiannis, Josephine M. Egan

**Affiliations:** Laboratory of Clinical Investigation, NIA-NIH, 251 Bayview Blvd, Baltimore, MD 21224, USA

**Keywords:** amyloids, Alzheimer’s disease, diabetes, biomarkers, evolution, alternative splicing

## Abstract

(1) Background and aims: Amyloidosis due to aggregation of amyloid-β (Aβ_42_) is a key pathogenic event in Alzheimer’s disease (AD), whereas aggregation of mature islet amyloid polypeptide (IAPP_37_) in human islets leads to β-cell dysfunction. The aim of this study is to uncover potential biomarkers that might additionally point to therapy for early AD patients. (2) Methods: We used bioinformatic approach to uncover novel IAPP isoforms and developed a quantitative selective reaction monitoring (SRM) proteomic assay to measure their peptide levels in human plasma and CSF from individuals with early AD and controls, as well as postmortem cerebrum of clinical confirmed AD and controls. We used Thioflavin T amyloid reporter assay to measure the IAPP isoform fibrillation propensity and anti-amyloid potential against aggregation of Aβ_42_ and IAPP_37_. (3) Results: We uncovered hominid-specific IAPP isoforms: hIAPPβ, which encodes an elongated propeptide, and hIAPPγ, which is processed to mature IAPP_25_ instead of IAPP_37_. We found that hIAPPβ was significantly reduced in the plasma of AD patients with the accuracy of 89%. We uncovered that IAPP_25_ and a GDNF derived DNSP_11_ were nonaggregating peptides that inhibited the aggregation of IAPP_37_ and Aβ_42_. (4) Conclusions: The novel peptides derived from hIAPP isoforms have potential to serve as blood-derived biomarkers for early AD and be developed as peptide based anti-amyloid medicine.

## 1. Introduction

Human islet amyloid polypeptide (IAPP), a member of the calcitonin-like gene family, is co-released with insulin from pancreatic β-cells upon glucose stimulation. It forms toxic β-sheets, oligomers, and fibrils in cytoplasm and around β-cells, thereby contributing to β-cell dysfunction [1,2]. The amyloidogenic propensity of primate IAPP is an evolutionary conundrum, since IAPP does not form fibrils in some mammalian species that lack the core amyloid sequences, while it does so in other species [3]. The quantity of macromolecules involved in various proteostasis mechanisms (i.e., control of membrane IAPP trafficking, proteosome degradation, endoplasmic reticulum unfolded protein response (UPR), and autophagy) [4] are drastically reduced in β-cells compared to other islet cell types [5], since insulin and IAPP account for more than one-half of their mRNA and protein content [6,7]. Therefore, human evolution adaptation may have led to additional intra- and extracellular chaperones to counter the proteinopathies of β-cells. Proinsulin and insulin are known inhibitors of IAPP aggregation [8], and we recently found that a short 19-AA Cα-peptide derived from the 74-AA proinsulin isoform inhibited fibrillation of IAPP_37_ [9]. In this investigation, we report additional peptide chaperones, a nonaggregating IAPP_25_ from β-cells, and a DNSP_11_ from α-cells that inhibit fibrillation of human mature IAPP_37_ and Aβ_42_.

Human 89-AA preproIAPP undergoes extensive posttranslational modification: cleavage of a 22-AA signal peptide and formation of a disulfide bond between Cys-2 and -7 in the endoplasmic reticulum resulting in 67-AA proIAPP that is transported to the Golgi apparatus and then packaged in secretory granules (SG); it is further processed, similar to proinsulin processing, to remove an 11-AA proIAPP_11_ peptide from the N-terminus by prohormone convertase 2 (PC2) and a 16-AA peptide from the C-terminus by PC1/3 [10]. The Lys-Arg AAs at the C-terminus are then removed by carboxypeptidase E, and peptidylglycine α-amidating monooxygenase (PAM) amidates the remaining glycine that is then converted to an amide group at the C-terminus of IAPP_37_ and release of glyoxylate [11]. The resulting mature IAPP_37_ is primarily localized in the halo of secretory granules (SG) (the pale outer area of β-cell SG seen on electron microscopic images) alongside C-peptide, while insulin itself is in the dense core of SG in β-cells [12].

We hypothesized that chaperones secreted from healthy α- and β-cells could counter IAPP_37_ aggregation in human islets. Glial cell-derived neurotrophic factor (GDNF), which is protective to dopaminergic neurons [13], is enriched in pancreatic islets [14] and is actually expressed at higher levels in the islets than in the brain [15]. Mwangi et al. found that GDNF can enhance β-cell proliferation and mass [16,17], whereas Lucini et al. found that GDNF colocalized only with glucagon-expressing α-cells in the pancreas [18]. The pro-peptide of a longer GDNFα isoform is cleaved by prohormone convertases to produce an amidated dopamine neuron stimulating peptide containing 11-AA (DNSP_11_; PPEAPAEDRSL) that is protective to dopaminergic neurons in animal models of Parkinson’s disease [19,20,21]. However, there is no report of DNSP_11_ inhibiting IAPP_37_ aggregation. Therefore, we carried out a ThT amyloid inhibitory assay with the novel hIAPP_25_ and the known DNSP_11_ in this study.

The common feature of T2DM and AD is progressive self-aggregation of an amyloidogenic protein, predominantly IAPP_37_ in islets and Aβ in brain regions. However, IAPP_37_ fibrils also deposit in cerebral vessels [22] and cross-seed with Aβ during the formation of amyloid plaques [23,24]. Also pointing to a relationship between AD and T2DM, plasma IAPP (and insulin) levels are reduced in mild cognitive impairment (MCI) and AD patients [25,26] and in the later stages of T2DM [27]. A nonaggregating synthetic IAPP, pramlintide, is reported to improve cognition in AD patients and reduce Aβ and phospho-Tau load in the cortex and hippocampus of 5XFAD transgenic mice [28,29]. Flavonoid silybins also inhibit aggregation of both IAPP and Aβ [30,31]. The evidence reinforces the notion that an association between AD and T2DM may rely on the cross-seeding properties of IAPP_37_ and Aβ [32,33]. In the present study, using a quantitative SRM proteomic assay, we identified and quantified the translated protein levels of two novel IAPP isoforms: hIAPPβ, which was increased in the cerebrum and reduced in the plasma of AD patients, and hIAPPγ, which encodes a novel mature IAPP that is 25-AA in length, is nonamyloidogenic, and was found to be reduced in T2DM islets and in the plasma of AD patients.

## 2. Materials and Methods

### 2.1. Source of Tissue Samples

Islets from human nondiabetic and T2DM postmortem pancreata [9] were provided by the NIDDK-funded Integrated Islet Distribution Program (IIDP) at City of Hope, NIH Grant # 2UC4DK098085 (https://iidp.coh.org/ (accessed on 13 July 2017, to 8 April 2019)). A total of 1000–2000 islets were received for each sample, and approximately 200 islets were handpicked for proteomics and the rest for gene expression experiments [9]. Human tissue total RNA was obtained from TaKaRa Bio (Takara Bio USA, San Jose, CA, USA). Plasma and cerebrospinal fluid (CSF) samples were from the IRB-approved study of NCT01255163 (https://clinicaltrials.gov/ct2/show/NCT01255163 (accessed on 18 November 2016)) [34].

### 2.2. Participants

We tested the diagnostic performance of the SRM assay for hIAPPβ and hIAPPγ in a cohort of 10 individuals with high-probability early AD according to the NIA-AA and IWG-2 criteria [35] and 19 cognitively normal healthy controls. Individuals with AD had abnormal CSF levels of amyloid β-peptide (Aβ) 1–42 (Aβ42) < 192 pg/mL and of P-T181-tau > 23 pg/mL that supported their high-probability diagnosis [36]. All participants were evaluated at the Clinical Research Unit of the U.S. National Institute on Aging (NIA; Baltimore, MD, USA) under NIH IRP approved protocols.

### 2.3. Bioinformatics and Sanger Sequencing

We used Sequencher 5.4.6 software (Gene Codes Corporation, Ann Arbor, MI, USA) to assemble pancreatic islet and insulinoma EST sequences (http://www.ncbi.nlm.nih.gov/dbEST/ (accessed on 24 July 2017)) that are homologous to the human *IAPP* gene. IAPP sequences from different species were downloaded from Ensembl (https://useast.ensembl.org/index.html (accessed on 26 July 2017)) and UCSC (https://genome.ucsc.edu/ (accessed on 26 July 2017)) genome browsers. The ExPASy server was used to analyze peptide properties [37], and EMBL-EBI (https://www.ebi.ac.uk (accessed on 26 July 2017)) was used for protein sequence alignments. The IMAGE EST clones IMAG6132753, IMAG6028075, and IMAG6218226 were ordered from Source Bioscience (https://www.sourcebioscience.com/ (accessed on 16 October 2017)), and the inserts were sequenced bidirectionally by the Sanger sequencing service of Eurofins Genomics (Louisville, KY, USA).

### 2.4. RNA Isolation, cDNA Synthesis, RT-qPCR, and ELISA

RNA isolation, cDNA synthesis, and RT-qPCR procedures were described previously [38]. We designed splicing junction specific TaqMan probes (Table S1) for IAPP isoforms that were custom made by Thermo Fisher Scientific (Waltham, MA, USA). The duplex fluorescent TaqMan assay was performed in replicates (StepOnePlus^TM^ real-time PCR system), and the relative fold change was calculated using the Formula (2)^(−ΔΔCt) normalized to GAPDH (vic-labeled, Cat# 4326317E) [39]. Droplet digital PCR (ddPCR) absolute values were derived from the Poisson distribution of positive and negative droplets (QX200 ddPCR System (Bio-Rad, Philadelphia, PA, USA), and fam-IAPP isoform-specific droplets were normalized with those of vic-GAPDH. We used 16 AD and 16 non-AD MTG samples and 8 T2DM (fresh) and 15 normal control (13 fresh and 2 frozen) islet samples for ddPCR assay. Plasma IAPP was quantified using a Human IAPP ELISA Kit (Cat# LS-F9686, LSBio, Seattle, WA, USA).

### 2.5. Selected Reaction Monitoring (SRM)-MS Assay

The selected peptides (Table S2) were synthesized as synthetic steady-isotope-labeled (heavy) standard peptide analogs and unlabeled analogs (light) by Genemed Synthesis Inc. (San Antonio, TX, USA). Details of SRM parameters, linear range of quantification, and limit of quantification (LOQ) followed our previous protocol [9]. All sample data generated from the islet (freshly handpicked ~200 islets per donor of T2DM 8 individual donors and control 8 individual donors; 1 in T2DM and 3 in control islet samples did not have SRM data), brain middle temporal gyrus (MTG) (250 µg), plasma (5 μL), and CSF (250 μL) samples were collected using Analyst software version 1.6.3 and processed using MultiQuant software (Sciex, version 3.02 with Scheduled-MRM-Algorithm). Each peak area was manually inspected to ensure correct peak detection and accurate integration. The relative quantitation value of each given peptide was obtained by summing all peak area ratios of the light per heavy from all target transitions of this peptide and then averaging over three technical runs. 

### 2.6. Thioflavin T In Vitro Assay for IAPP and Aβ Amyloid Formation

Thioflavin T (ThT) was purchased from Millipore-Sigma (Cat# T3516, Rockville, MD, USA). The APP_37_ peptide (37-AA) (Cat# AS-60254-1, amidated C-terminal, and disulfide bridge between Cys2 and Cys7), Aβ_42_ (1–42) (Cat# AS-20276), and C-peptide (Cat# AS-61127) were from AnaSpec (Fremont, CA, USA). Black 96-well microplates (Chimney Well) were obtained from Greiner Bio-One (Frickenhausen, Germany). C-terminal amidated IAPP_25_, DNSP_11_, Cα (EAEDLQGSLQPLALEGSLQ), and INSU (MGSETIKPAGAQQPSALQDRLHQKRPSSRSLSFCHGPVDAPPAPAGAAGPLGT) derived from human *INS* upstream open reading frame peptides [9] were synthesized by GeneMed Synthesis Inc. (San Antonio, TX, USA). Monomeric IAPP_37_ and Aβ_42_ were made in hexafluoroisopropanol (HFIP, Cat# 105228, Sigma-Aldrich, St. Louis, MO, USA) solution and then lyophilized. The final concentration of the monomeric peptides was 1% DMSO and 25 μM ThT in Component A buffer (AnaSpec Manual of SensoLyte Thioflavin T Aggregation Kit, Cat# AS-72214). ThT buffer was used as a blank, and 50 μM IAPP_37_ or Aβ_42_ without inhibitors was used as a control. The final concentrations of IAPP_37_ and Aβ_42_ with the inhibitory IAPP_25_, DNSP_11_, Cα, and INSU peptides were 50 μM in the fibrillation kinetic assays [9]. Aggregation was measured by increasing the ThT fluorescent intensity (λex = 440 nm, λem = 485 nm, height 3 mm, flashes 12) for 36 cycles for IAPP_37_ and Aβ_42_, 5 min per cycle at 37 °C with 15 s of shaking (100 rpm) between the reads in an EnSpire Multimode Plate Reader (PerkinElmer Inc., Waltham, MA, USA).

### 2.7. Statistical Data Analysis

GraphPad Prism v9 software was used for statistical analysis of RT-qPCR and SRM data. *p* < 0.05 was considered significant, and data are represented as the means ± SEM. The normalized expression values of hIAPP isoforms in TaqMan fold changes and ddPCR IAPP/GAPDH droplets, ELISA, and SRM quantitative values were analyzed with unpaired Student’s *t*-test and the Pearson correlation coefficient matrix. Biomarker accuracies were calculated by the area under the receiver operating characteristic curve (AUROC). For the ThT amyloid dye assay, the area under the curve (AUC) was calculated using the trapezoidal rule [40], and the data were analyzed using one-way ANOVA with Tukey’s multiple comparisons [41].

## 3. Results

### 3.1. Novel Hominid-Specific Peptides Derived from hIAPP Isoforms as Potential Diagnostic and Therapeutic Targets for AD and T2DM

#### 3.1.1. Identification of hIAPPβ and hIAPPγ Isoforms

**Figure 1:**
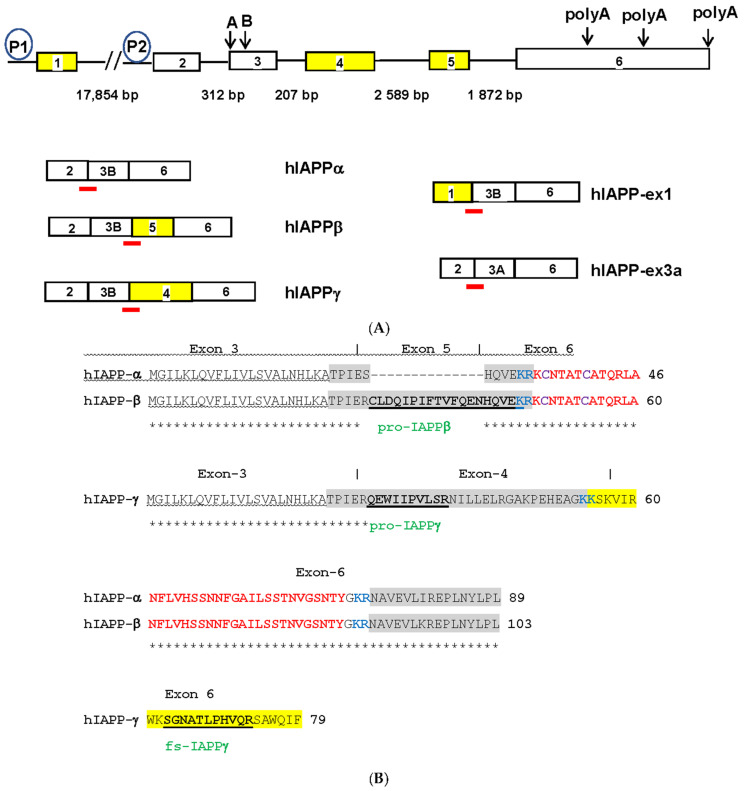

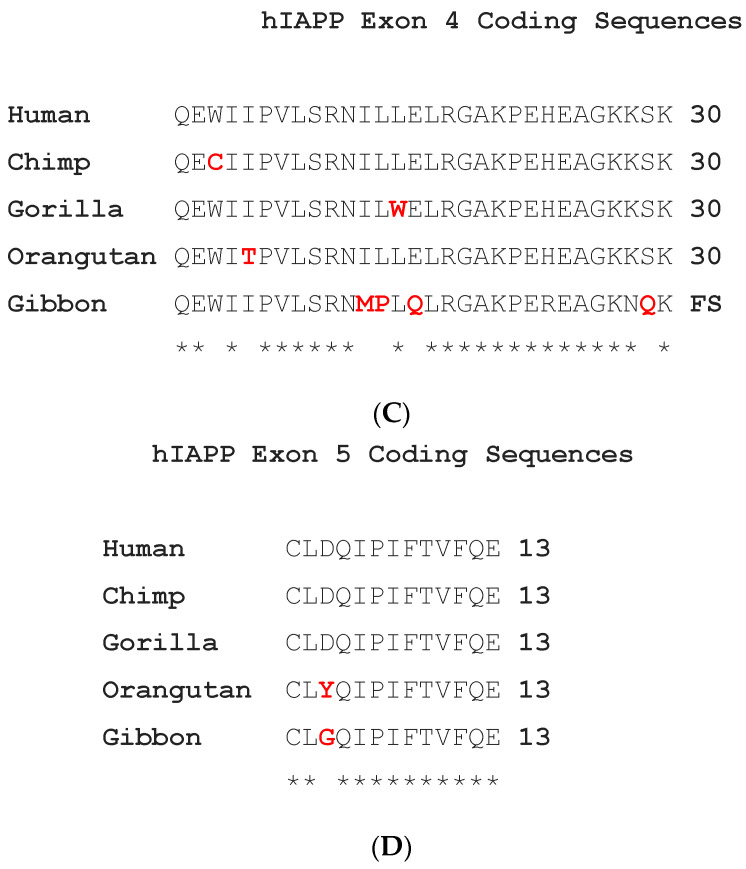
(**A**) Human *IAPP* gene structures and alternatively spliced isoforms. Open boxes represent exons, solid lines represent introns of different sizes in base pairs (bp) and P1 and P2 in circles are alternative promoters. Yellow boxes represent hominid-specific IAPP exons. Downward arrows and capital letters are at the intra-exonal splicing donor sites in exon 3 and alternative polyadenylation sites in exon 6. The red bars are exon junctional TaqMan probes that hybridize specifically to various IAPP isoforms. The spliced transcripts of hIAPP isoforms are named and below the gene structure. hIAPP-ex1 and -ex3a are not expressed and are expressed at low levels in islets, respectively. (**B**) Alignment of hIAPPα, hIAPPβ, and hIAPPγ amino acid sequences. Homologous amino acids are marked by asterisks, signal peptide underlined by wavy lines, prohormone regions by gray highlight, the convertase dibasic amino acids by blue lettering, mature IAPP_37_ by red lettering, bisulfide bond cysteine (**C**) by purple lettering, IAPP_25_ by yellow highlight, the proteolytic peptide names by green lettering, and the sequences are bolded and underlined. Exon numbers are above the sequences and demarcated by the **|** sign. (**C**) Alignment of exon 4 of the hIAPPγ CDS. (**D**) Alignment of exon 5 of the hIAPPβ CDS. The amino acids coded by nonsynonymous substitutions are shown in red lettering.

We searched dbEST sequences [42] and found 336 ESTs that were homologous to human IAPP mRNA (NM_000415). Assembly of the ESTs revealed additional protein coding IAPP isoforms of four exons instead of the conventional three exons [43]. We uncovered novel IAPP isoforms composed of four exons (Figure 1A): hIAPPβ contains an alternatively spliced exon 5 translated into a longer prohormone adding 14-AA to prototype proIAPP_11_ of hIAPPα, while hIAPPγ contains an alternatively spliced exon 4 translated into an unrelated proIAPP_32_ (only 4-AA at the N-terminus conserved to proIAPP_11_ of hIAPPα) and a frameshifted IAPP_25_ completely different from IAPP_37_ (Figure 1B). The coding sequences (CDSs) of orthologs of novel IAPP exon 4 were only found in hominid (Figure 1C), except for the gibbon ortholog that contains a single nucleotide deletion (Figure S1A), and the orthologs of other primate species contain nucleotide insertions causing frameshifts (Figure S1B). There is one nonsynonymous substitution (Trp/Cys) between human and chimp, indicating adaptive evolution (positive selection) of hIAPPγ. The CDS of orthologs of exon 5 was found in hominid and not in old-world monkeys, except for a single nucleotide deletion in *Chlorocebus sabaeus* and orthologs of lemurs and marmoset, two small primates evolving separately in Madagascar and South America. There are no nonsynonymous or synonymous substitutions of exon 5 among humans, chimp, and gorillas (Figure S1C), indicating neutral selection of hIAPPβ in humans. The sequences of all hIAPP coding exons are identical to those of the Neanderthal genome [44].

hIAPPγ does not contain the C-terminal prohormone region of hIAPPα and is processed to a 25-AA peptide, IAPP_25_, by prohormone convertase (at paired basic amino acid KK in Figure 1B). IAPP_25_ (KSKVIRWKSGNATLPHVQRSAWQIF) does not contain any amyloid core sequences or consensus sites for proteases at the C-terminus [45], nor does it have any homology with any protein domains, families, or functional sites in the PROSITE database (https://prosite.expasy.org/ (accessed on 31 January 2018)). It has a theoretical molecular weight (MW) of 2938 Daltons (Dal) and an isoelectric point (pI) of 12.0, which is more basic than IAPP_37_ (MW = 3906 Dal, pI = 8.9). We revised the human IAPP genomic structure to six exons, of which exon 6 is a constitute exon with three alternative polyadenylation sites at its 3′UTR. Exon 1 (NM_001329201) and exon 2 are alternative promoter initiation exons, and exon 3 has two intra-exonal splicing sites, including the exon 3A (IAPP-ex3A, BM876150) splicing site 20 bp upstream of conventional exon 3B. IAPP-ex3A isoform expression was approximately 20-fold lower than hIAPPα and hIAPPγ (exon 3B splicing site) expression in islets. IAPP-ex1 was conserved only in human, chimp, and gorilla (Figure S1D) and could be detected in testes instead of islets (Figure S2A). To validate the novel IAPP isoforms, we sequenced EST clones of IMAG6132753 (CA771728) and IMAG6028075 (BQ548915) and found that they were full-length cDNA clones of hIAPPα and hIAPPγ, respectively. The EST clone IMAG6218226 of hIAPPβ (CA774544) is not commercially available, so we used mass spectrometry (MS)-based SRM to validate it at the protein level.

#### 3.1.2. Validation and Quantification of hIAPPβ and hIAPPγ by MS-Based SRM Assay

**Figure 2:**
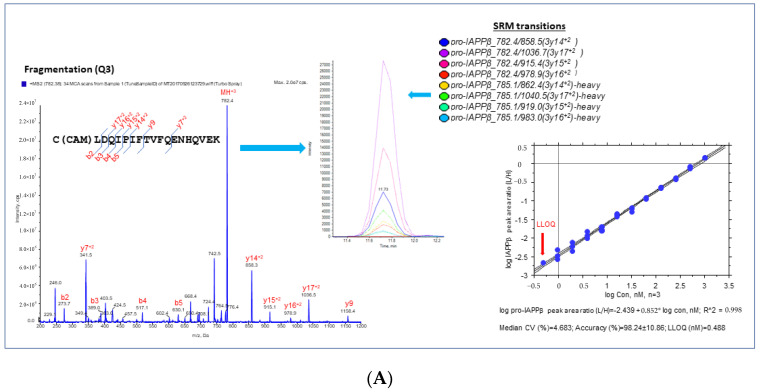

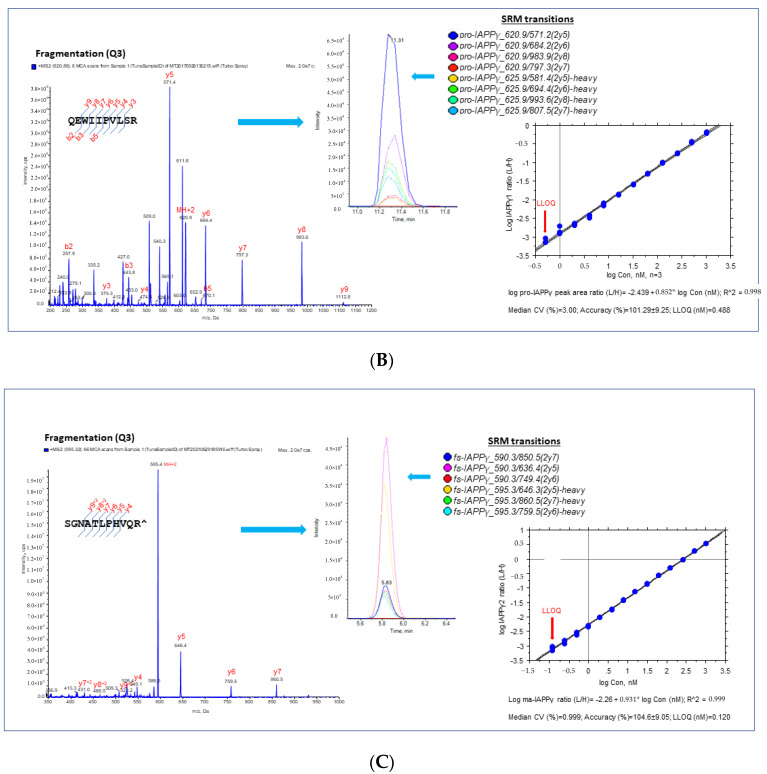
Proteotypic peptides for SRM assay of IAPPs. MS/MS spectra generated by SRM reaction of transitions with light or heavy SIS of target peptides, pro-IAPPβ (**A**), pro-IAPPγ (**B**), and fs-IAPPγ (**C**) in the (**left panels**). A set of coeluting peaks in the plasma matrix confirms that the detected SRM signals are derived from each proteotypic peptide in the (**middle panels**). The quantitative validation of the SRM assay is displayed in the (**right panels**). The calibration curve of the SRM assay for each proteotypic peptide in the plasma matrix was carried out as follows: (1) linearity was determined by linear regression between the measured peak area ratio of light and heavy SIS versus the theoretical concentration of light-SIS peptide in the plasma matrix prepared from pooled samples; we display the linear regression with the dashed line representing CI (95%) for the mean, *n* = 3; (2) accuracy was estimated by back fitting data to the STD curve from all quantified points (>5 points) in the plots, as expressed as the mean ± SD (%); and (3) LLOQ was determined from the standard curve, defined as the lowest concentration with acceptable CV < 20% and accuracy within 100 ± 20%.

We selected proteotypic peptides based on the empirical procedure that balances ideal attributes of the assays with practical limitations, e.g., hominid-specific CDS of the exon 5 region (pro-IAPPβ) and the CDS of exon 4 region (pro-IAPPγ) and the frameshifted (fs) mature peptide region (fs-IAPPγ) (Figure 1B). Interference-free precursors and fragments (transitions) for target proteins constitute the final SRM assay, i.e., four transition pairs for pro-IAPPβ (Figure 2A), four transition pairs for pro-IAPPγ (Figure 2B), and three transition pairs for fs-IAPPγ (Figure 2C). All proteotypic peptides for the hIAPP isoform SRM assay, calibration graphs with linear fitting, correlation coefficient R^2^, and LOQ are shown in the right panel of Figure 2.

We found that pro-IAPPγ and fs-IAPPγ were at similar levels but only approximately 20% of the level of pro-IAPPβ in plasma (Figure S2B). We performed a Pearson correlation analysis between IAPP by IAPP-ELISA, which detects IAPP_37_ only, and SRM results and found significant correlations of IAPP-ELISA with pro-IAPPβ (r = 0.43, *p* = 0.02) but not with pro-IAPPγ (r = 0.03, *p* = 0.87) or fs-IAPPγ (r = 0.01, *p* = 0.95) in the plasma samples (*n* = 28) (Figure S3A). We concluded that hIAPPγ is not recognized in plasma by ELISA for IAPP, perhaps because the antibody is specific for mature IAPP_37_.

#### 3.1.3. hIAPPβ and hIAPPγ in T2DM Islets and AD Cerebrum

**Figure 3:**
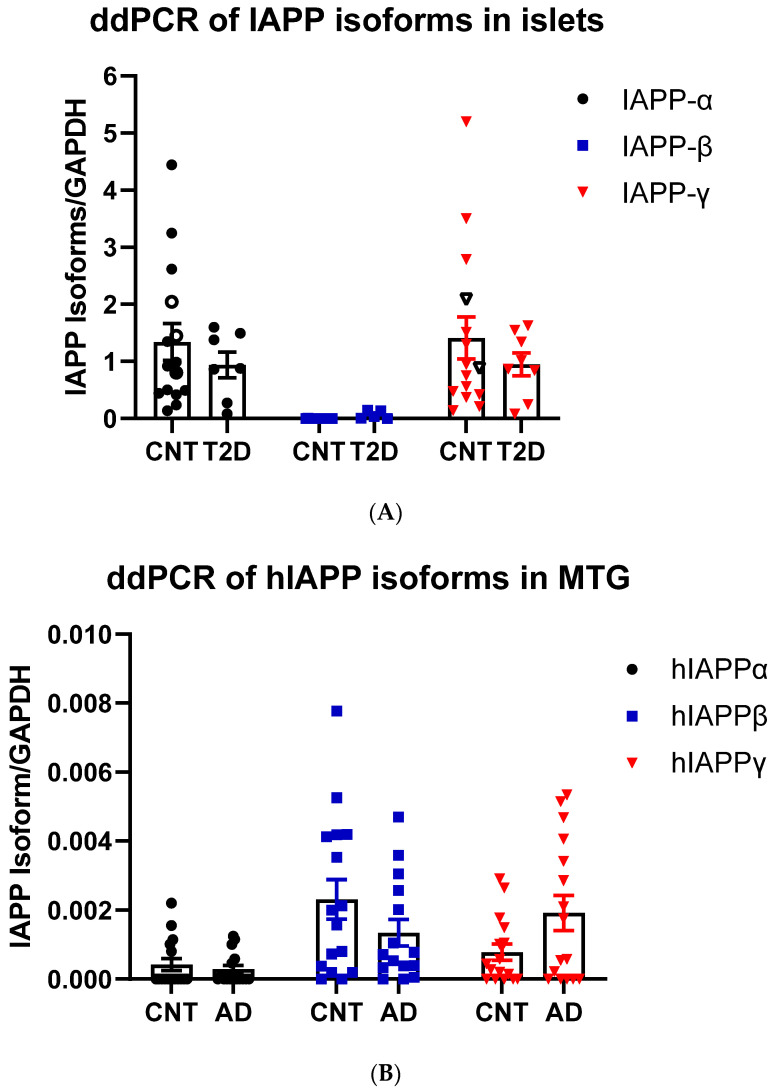

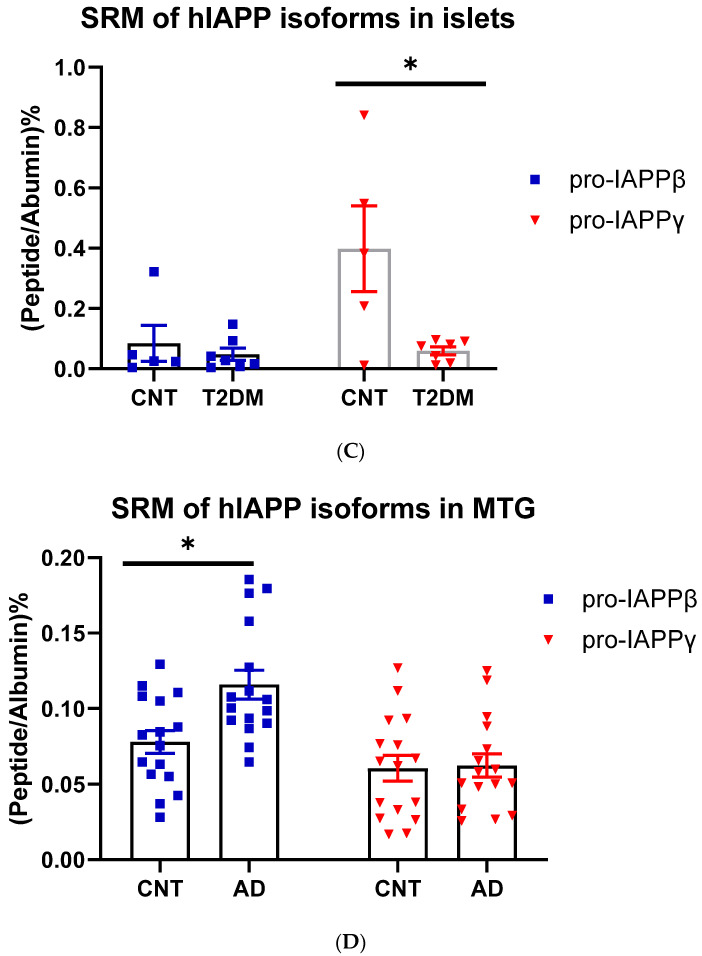
IAPP isoform expression in islets and cerebrum. (**A**) ddPCR of IAPP isoform expression in islets of controls (CNT, *n* = 14–15, 12–13 fresh islets of close marks and two frozen of open marks) and type 2 diabetes (T2DM, *n* = 7–8) and (**B**) in the MTG of controls (CNT, *n* = 15–16) and Alzheimer’s disease (AD, *n* = 15–16). The Y-axis shows droplets of islet IAPP isoforms normalized to those of GAPDH. (**C**) SRM measurements of pro-IAPPβ and pro-IAPPγ at the peptide level in islets of controls (CNT, *n* = 5) and type 2 diabetes (T2DM, *n* = 7) and (**D**) in the MTG of controls (CNT, *n* = 16) and Alzheimer’s disease (AD, *n* = 16). The Y-axis is the percentage of IAPP isoform peptides normalized to albumin. * represents statistically significant (*p* < 0.05).

We designed TaqMan probes at splicing junctions that specifically hybridize to hIAPPα, hIAPPβ, and hIAPPγ (Figure 1A and Table S1) isoforms and compared their expression in nondiabetic and T2DM islets and non-AD and AD middle temporal gyrus (MTG) that has Aβ burden early in the disease [46] using a sensitive ddPCR method. As expected, hIAPPα was highly expressed in islets, and we also found that hIAPPγ expression was at a similar level, while hIAPPβ mRNA was approximately 0.1% of hIAPPα (Figure 3A). We were able to detect mRNAs of hIAPPα (5 out of 15), hIAPPβ (14 out of 16), and hIAPPγ (11 out of 16) at low levels in MTG samples (Figure 3B) using ddPCR. Islet hIAPPα and hIAPPγ expression levels were 2990- and 1800-fold higher than those of MTG, respectively. However, hIAPPβ mRNA level in islets was 44% that of MTG and it was 6-fold higher than that of hIAPPα in human MTG. There were no significant changes at the mRNA level in T2DM islets or AD MTG samples compared to those of controls (Figure 3A,B). At the protein level, pro-IAPPγ levels were reduced in T2DM islets to 15.9 ± 0.4% (t_(10)_ = 2.85, *p* = 0.017) of nondiabetic islets, while there were no differences in pro-IAPPβ levels (Figure 3C). In contrast, the pro-IAPPβ quantity was increased 1.27-fold (t_(30)_ = 3.11, *p* = 0.004) in the AD MTG compared to the non-AD samples (Figure 3D). We designed a peptide C(Cam)NTATC(Cam)ATQR that is located within IAPP_37_ for our SRM assay; unfortunately, its elution time was less than 2 min, and the transition peaks were not amenable to analyses. The change of IAPP isoform at protein levels in pathological islets and MTG prompted us to investigate whether they could serve as blood-based biomarkers in early AD patients.

#### 3.1.4. Reduction of hIAPPβ and hIAPPγ Peptides in AD Plasma Samples

**Figure 4:**
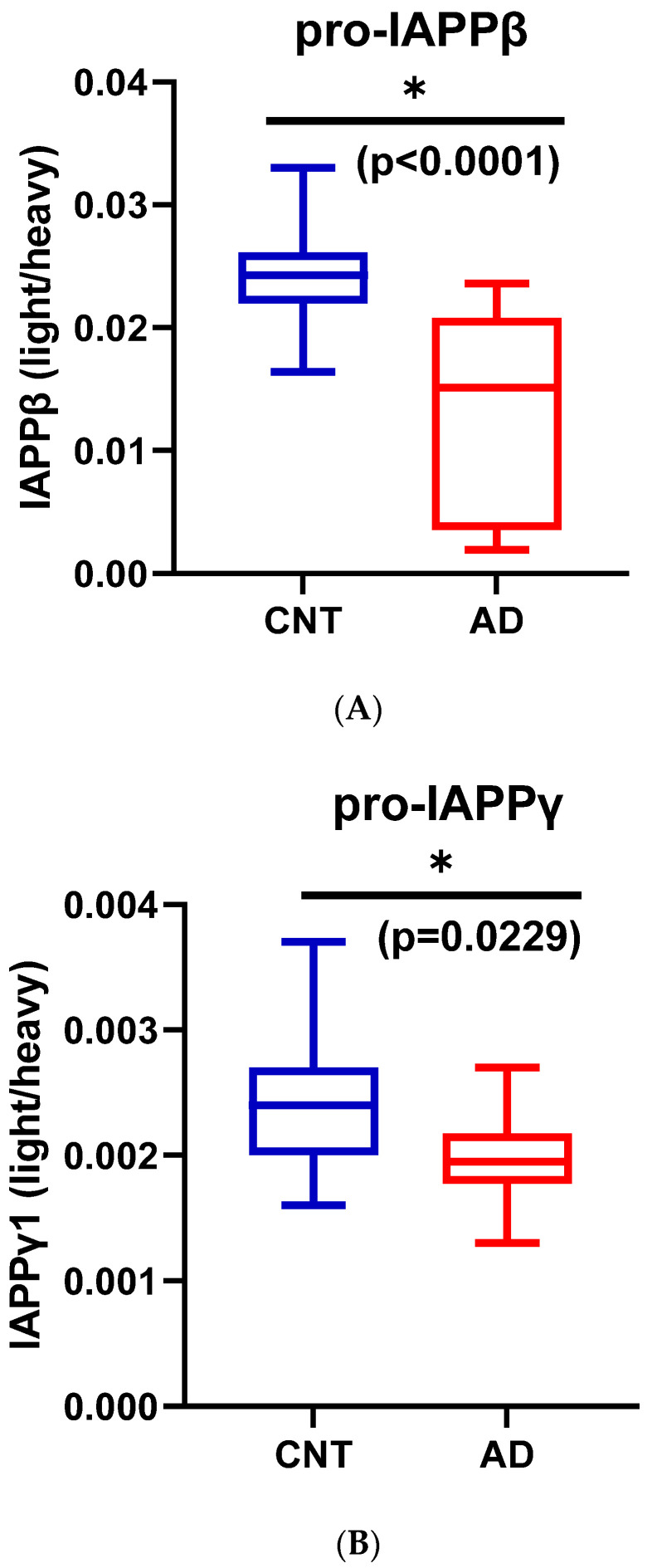

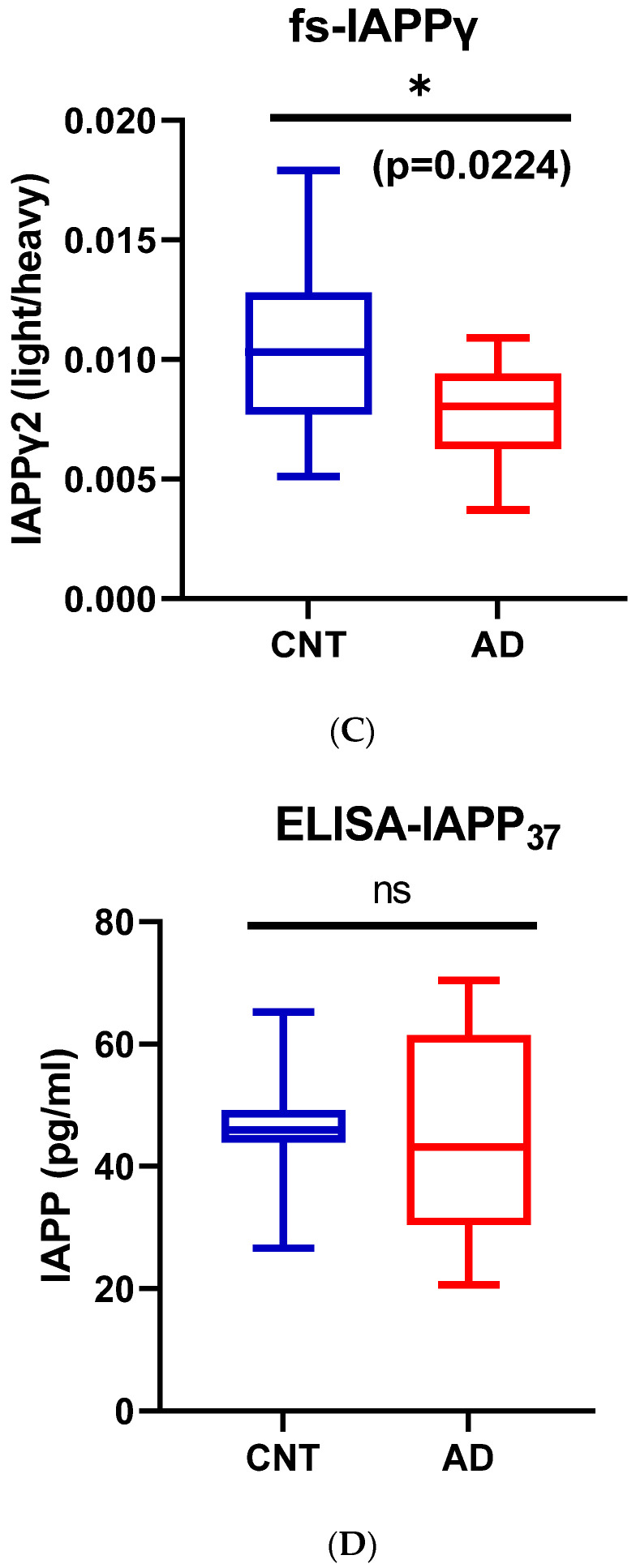

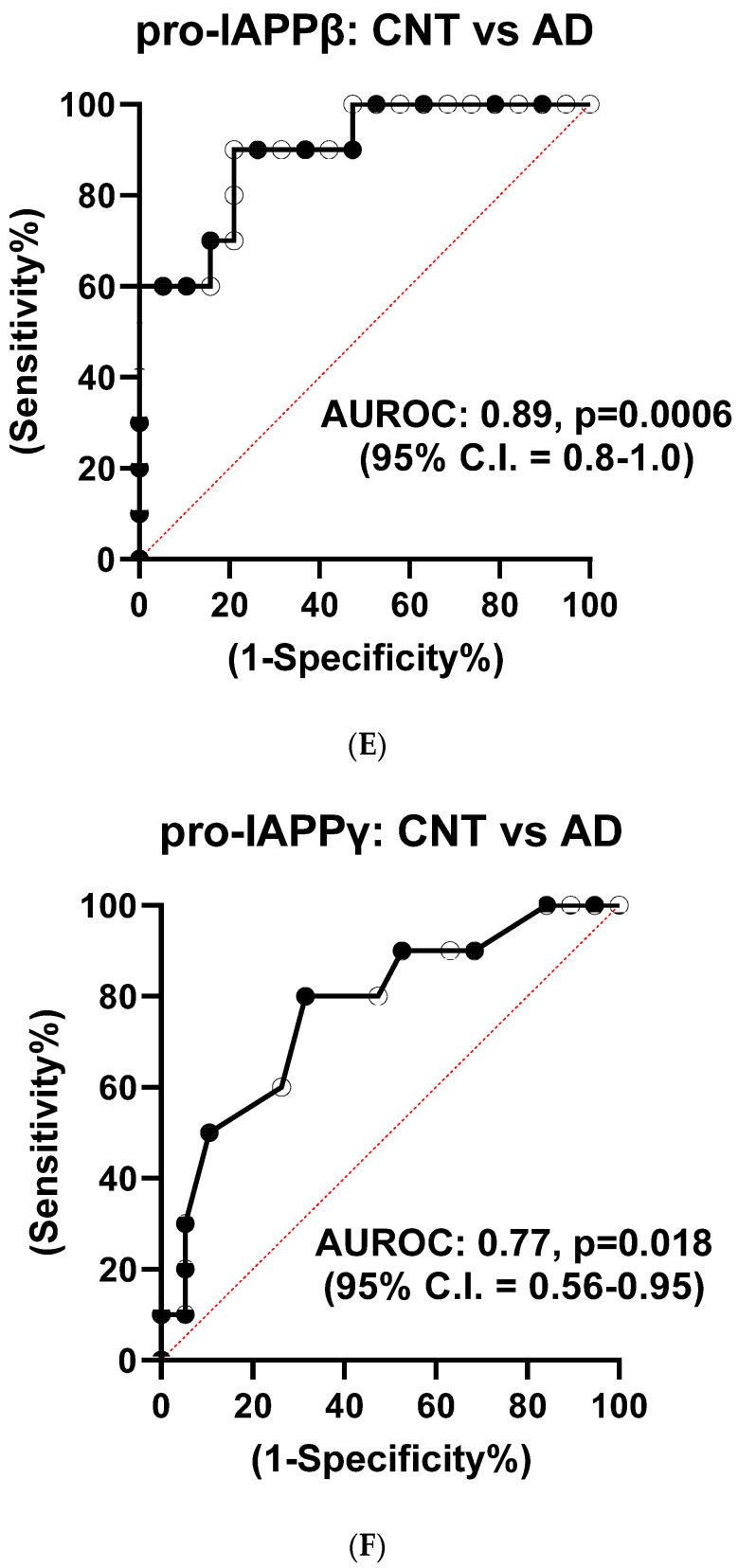

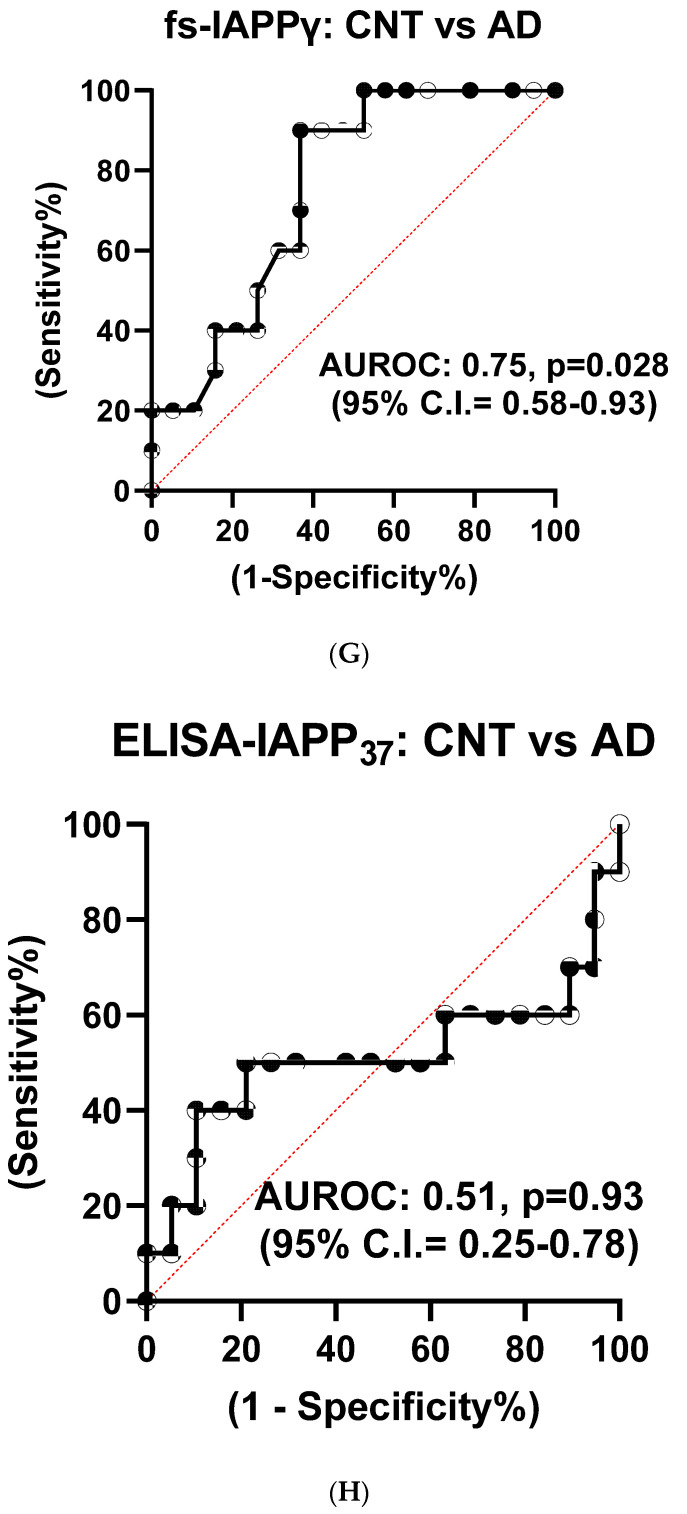
IAPP isoform peptide concentrations in control and AD plasma samples. Unpaired Student’s *t*-tests: (**A**) pro-IAPPβ; (**B**) pro-IAPPγ; (**C**) fs-IAPPγ; (**D**) IAPP-ELISA. The Y-axis is the ratio of peak area (light/heavy) for SRM and pg/mL for ELISA. Area under the receiver operating characteristic curve (AUROC) tests: (**E**) pro-IAPPβ; (**F**) pro-IAPPγ; (**G**) fs-IAPPγ; (**H**) IAPP-ELISA. The Y-axis is the true positive rate, and the X-axis is the true negative rate. ns: not significant.

We used partial least squares-discriminant analysis (PLS-DA) to reduce the number of variables in multidimensional SRM data and observed that the proportion of variance was centered in the first two components, i.e., component 1 (C1) explained 56.3% and component 2 (C2) explained 26.4% of the total variance (Figure S3B). Notably, pro-IAPPβ had the highest loading in C1 and C2. The first two components clearly separated the AD plasma samples from those of controls (Figure S3C). We found that the level of pro-IAPPβ was nine-fold and two-fold higher than that of pro-IAPPγ and fs-IAPPγ, respectively. The pro-IAPPβ, pro-IAPPγ, and fs-IAPPγ in AD plasma samples were reduced to 56.5% (t_(27)_ = 4.65, *p* < 0.0001), 82.0% (t_(27)_ = 2.41, *p* = 0.0229), and 72.6% (t_(27)_ = 2.42, *p* = 0.0224) of those in normal controls, respectively (Figure 4A–C), but we did not find significant differences (t_(26)_ = 0.25, *p* = 0.8044) by IAPP-ELISA (Figure 4D). We further evaluated the detection accuracy of hIAPP isoforms as plasma-based AD biomarkers using ROC analysis. We found that pro-IAPPβ, pro-IAPPγ, and fs-IAPPγ determined by SRM could effectively discriminate between AD and controls, with AUROC values of 0.89 (*p* = 0.0006), 0.77 (*p* = 0.018), and 0.75 (*p* = 0.028), respectively (Figure 4E–G), while the performance of the IAPP-ELISA was like chance, with an AUROC of 0.51 (*p* = 0.93) (Figure 4H). In CSF, the hIAPP isoforms were detectable but below the lower limit of quantification (LLOQ) using SRM.

#### 3.1.5. In Vitro Inhibition of IAPP_37_ and Ab_42_ Fibrillation by IAPP_25_ and DNSP_11_

**Figure 5:**
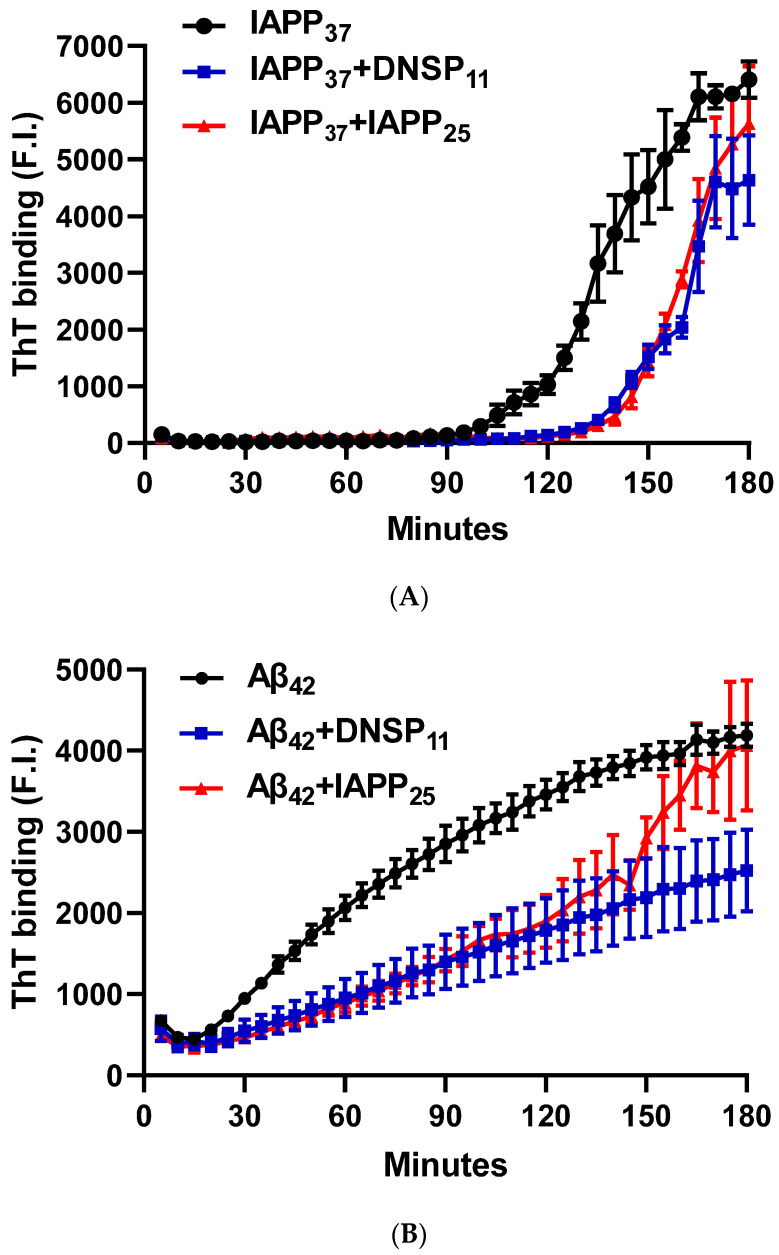

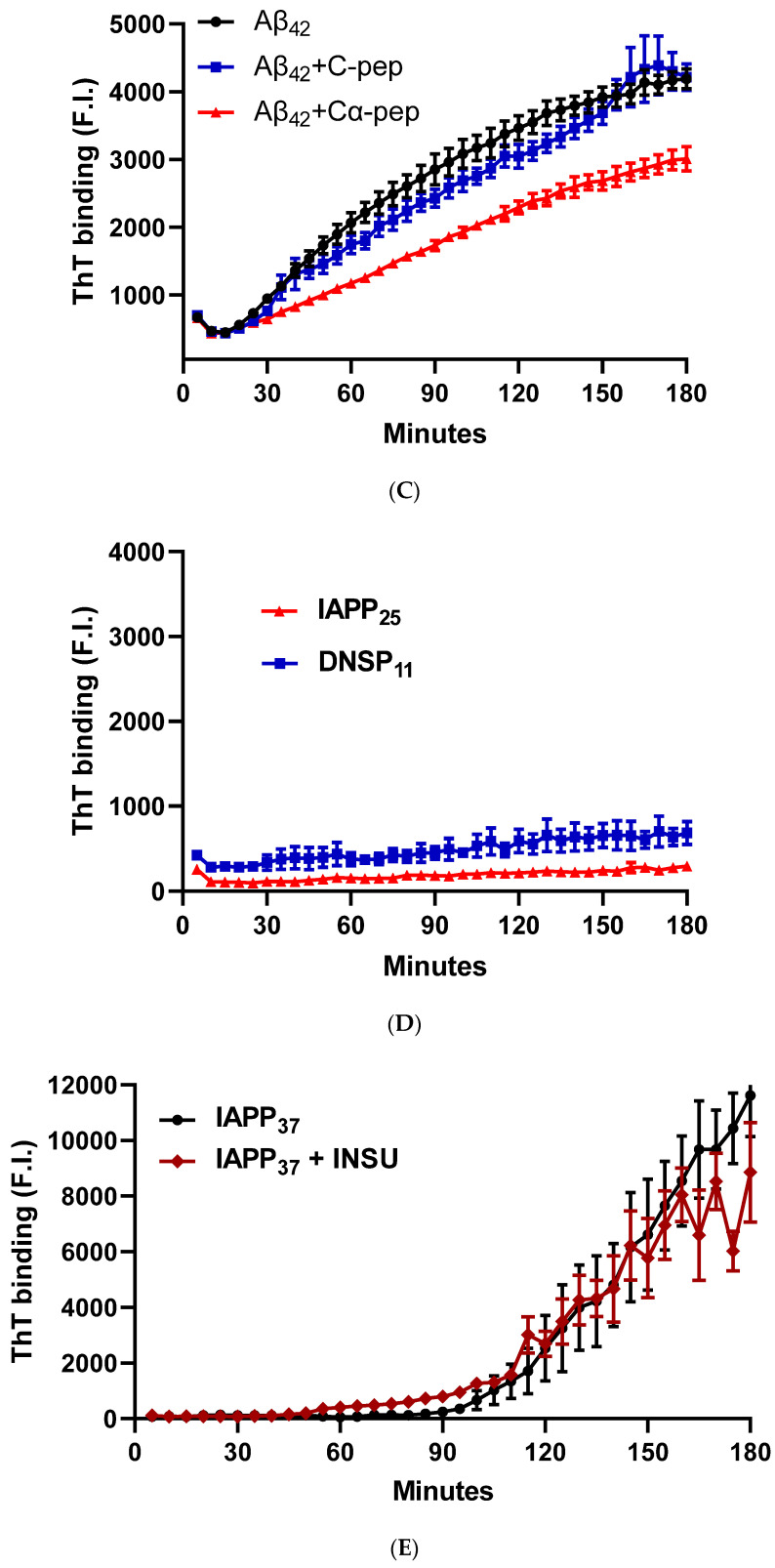

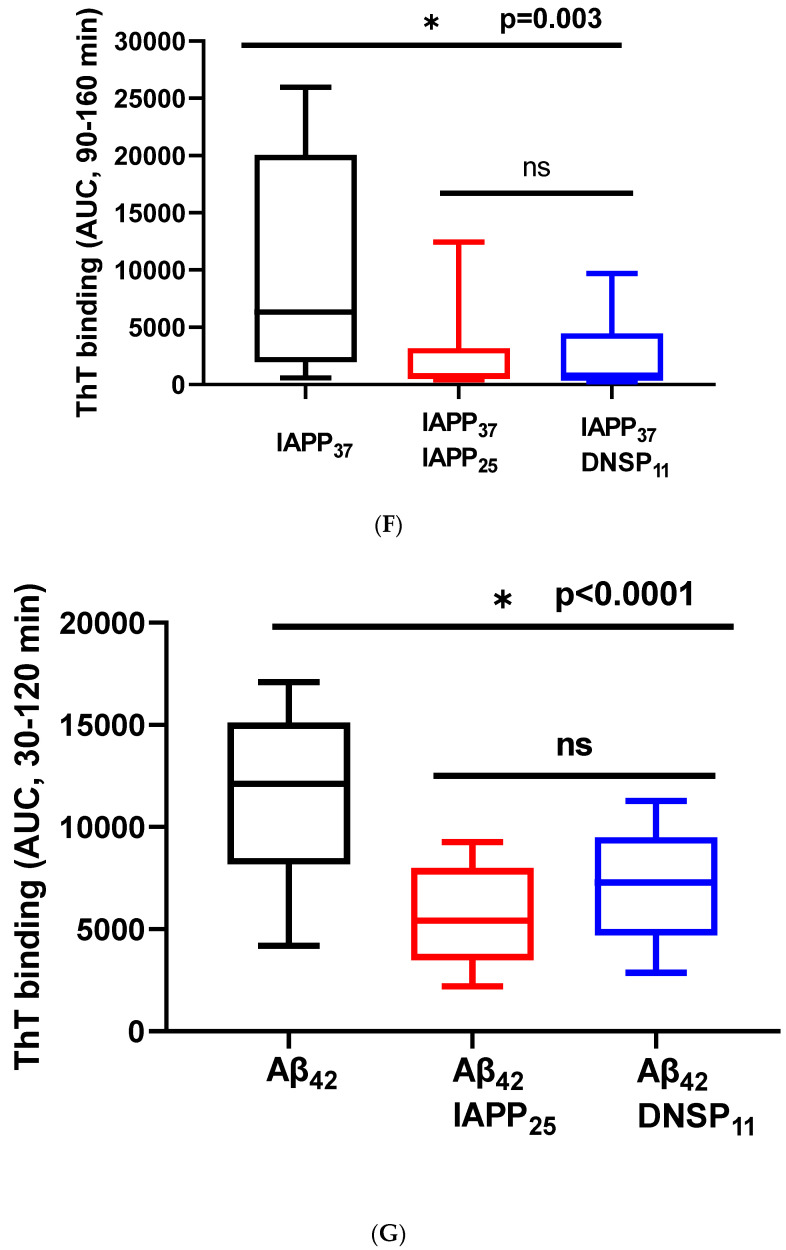

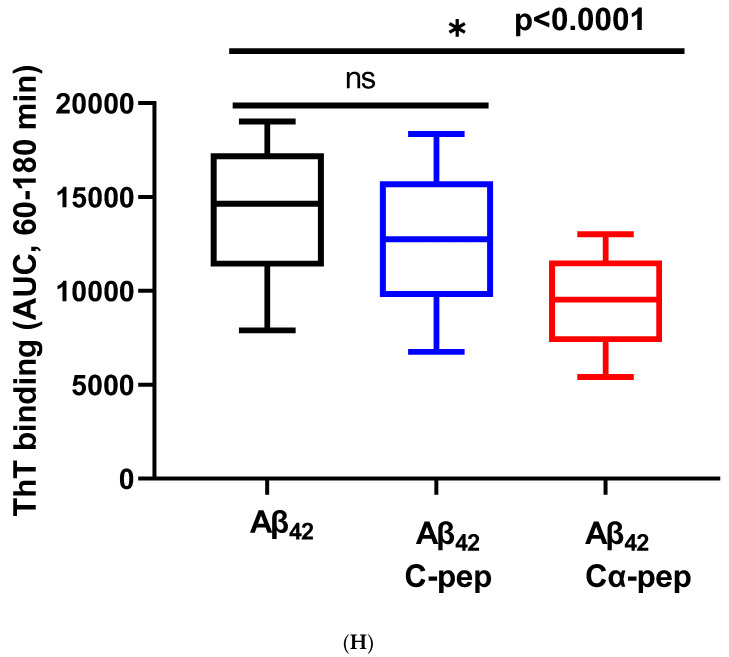
Inhibition of IAPP_37_ and Aβ_42_ fibrillation by IAPP_25_, DNSP_11_, and Cα-peptide. Y-axis represents the fluorescence intensity (F.I) of ThT binding and X-axis minutes. Representative fibrillation dynamics and inhibition curves in three replicates for (**A**) IAPP_37_ with equal molar concentration of IAPP_25_ and DNSP_11_; (**B**) Aβ_42_ with equal molar concentration of IAPP_25_ and DNSP_11_; (**C**) with equal molar concentration of of C- and Cα-peptides; (**D**) thioflavin T (ThT) amyloid reporter assay of IAPP_25_ and DNSP_11_; IAPP_25_ or DNSP_11_ alone did not bind to the ThT amyloid reporter. (**E**) No inhibition of IAPP_37_ fibrillation by insulin upstream open reading frame peptide occurs (INSU). Box-whisker plot of the inhibition. Y-axis is the area under the curve (AUC) of ThT fluorescence intensity. (**F**) Inhibition of IAPP_37_ fibrillation by IAPP_25_ and DNSP_11_ in the range of 90–160 min. (**G**) Inhibition of Aβ_42_ fibrillation by IAPP_25_ and by DNSP_11_ in the range of 30–120 min and (**H**) by C- and Cα-peptide in the range of 60–180 min. ns: not significant.

We tested whether DNSP_11_ and newly discovered IAPP_25_ peptide impacted Aβ_42_ and IAPP_37_ aggregation using Thioflavin T assay. Figure 5A is a representative experiment showing IAPP_37_ aggregation dynamics with a lag time of 90 min and inhibition by equal molar amounts of IAPP_25_ (derived from hIAPPγ) and DNSP_11_. We found that IAPP_37_ aggregation was inhibited by both IAPP_25_ and DNSP_11_ (25.0% and 24.1% of IAPP aggregation dynamics, respectively; *p* = 0.003) in the linear range of 90–160 min (Figure 5F). This finding implies that the reduction of IAPP_25_ in T2DM islets observed in Figure 3C might disinhibit IAPP fibrillation in T2DM islets, and DNSP_11_ might play a protective role in islets as it was shown in substantial nigra of brain [47].

The Aβ_42_ aggregation with a lag time of 20 min was also inhibited by equal molar amounts of IAPP_25_ and DNSP_11_ (Figure 5B) with 49.0 and 61.9% of Aβ aggregation dynamics, respectively, (*p* < 0.0001) in the linear range of 30–120 min (Figure 5G). The Aβ_42_ aggregation was inhibited by Cα-peptide (66.5% of Aβ_42_ aggregation dynamics) shown previously to inhibit IAPP_37_ fibril formation in vitro [9] but not inhibited by C-peptide (Figure 5C,H). IAPP_37_ aggregation was not affected by the equal molar amounts of the uORF of INSU peptide [9] used as a negative control (Figure 5D). All these suggest that equal molar ratio used in our experiments likely does not jeopardize the specificity of the observed inhibitory effects of DNSP_11_ and IAPP_25_.

## 4. Discussion

We uncovered two novel hominid-specific hIAPP isoforms derived from four exons instead of the conventional three exons. The hIAPPβ isoform has a 14-AA insertion in the N-terminal prohormone region of hIAPPα, but it is processed to the same mature IAPP_37_. The hIAPPγ isoform, on the other hand, has a different prohormone sequence from hIAPPα and a frameshifted preproIAPP that is processed to a nonaggregating peptide (IAPP_25_). *IAPP* belongs to a group of rapidly evolving genes known to have new primate-specific exons [48,49]. For example, coding sequences of exon 4 of hIAPPγ are conserved only in humans, Neanderthals, chimps, and gorillas, and exon 5 of hIAPPβ is conserved in hominids, including gibbons. The *IAPP* gene is one of the most human tissue-specific expressed genes, reportedly predominantly present in β-cells, although we found low levels of IAPP isoforms in human testes, including a hominid-specific upstream exon 1. hIAPPβ expression was higher in human cerebrum than in islets, resembling the chicken IAPP gene that is predominantly expressed in the brain [50], indicating that new exons might contribute to yet unknown neuroendocrine functions. On the other hand, hIAPPα and hIAPPγ expressions in islets are more than a thousand-fold higher than those in the MTG. That the hIAPPβ peptide level was higher in plasma than that of hIAPPγ implied the presence of hIAPPβ in gastrointestinal glands [51]. Direct comparison of SRM values of hIAPPα with hIAPPγ was hampered by the short elusion time (<2 min) of the amyloidogenic peptide for IAPP_37_ (CNTATCATQR) and the different absolute values of IAPP_25_ SRM and ELISA-IAPP_37_ in plasma [52]. SRM is supported by physical reactions that do not depend on antibody and epitope specificity. The LOQ differences of SRM and ELISA are more than 10-fold [53], so it is not possible to compare ELISA IAPP_37_ to SRM IAPP_25_ ratio in islets and plasma. We are currently developing IAPP_25_ antibody to compare IAPP_25_ and IAPP_37_ ratio with ELISA and Western Blot. Due to primate evolution favoring phenotypic longevity and intelligence, human-specific genome adaptation, either beneficial or adverse, is intimately associated with metabolic and tissue degenerative diseases. For instance, the human-specific APOE e3 allele arose approximately 220,000 and e2 approximately 80,000 years ago, perhaps mitigating any inherent cognitive adverse effects of the ancestral e4 allele [54] (also the major genetic risk factor for sporadic AD) [55,56]. In the same vein, new intra-exonal splicing of the human *INS* gene created the Cα-peptide that slows the rate of IAPP and Aβ fibrillation [9]; mutations of a primate specific gene ZNF808 cause neonatal diabetes due to pancreatic agenesis [57]; overexpression of human antisense transcripts of BDNF (BDNFOS) increases β-site APP cleaving enzyme 1 (BACE1) activity that can result in AD [15,58]; overexpression of human antisense transcripts of GDNF (GDNFOS1) occurs in AD brain [15] and unfortunately enhances the viability of glioblastoma cells [15,59], and upregulation of the human de novo gene FLJ33706 also occurs in the AD brain [60]. We now find that the hominid-specific hIAPPγ inhibits IAPP_37_ fibrillation; therefore, reduced hIAPPγ could accelerate IAPP aggregation in islets. On the other hand, increased hIAPPβ levels in the MTG leading to amyloidogenic IAPP_37_ could seed amyloidosis in the AD brain.

GDNF is highly expressed in α-cells of islets and is downregulated in AD brain regions [15,18]. GDNF sustains dopaminergic (DA) neurons and prevents their death in Parkinson’s disease (PD) [13]. DNSP_11_ derived from proGDNFα is localized in the substantia nigra and inhibits 6-hydroxydopamine-induced toxicity of dopamine neurons [19]. In addition, serum GDNF levels are significantly lower in T2DM [61]. However, there is no study on the role of DNSP_11_ in amyloid formation, whether α-synuclein is involved in Lewy body disease, Aβ is involved in AD, or IAPP is involved in islets in T2DM. Various peptides derived from IAPP, insulin, C-peptide, and chromogranin A have been developed to inhibit IAPP fibrillation [62].

Finally, it remains a challenge to develop cost-effective and noninvasive blood-based biomarkers to diagnose preclinical AD before extensive neuronal death occurs [63]. Previous studies by Adler et al. [25] and Zhu et al. [26] reported that IAPP_37_ is significantly reduced in AD plasma samples by ELISA, and our IAPP-ELISA did not reproduce their findings. The discrepancy of the SRM from the previous ELISA may be because our participants were a cohort with early AD symptoms. In this study, we used our SRM assay, achieving high diagnostic accuracy. This is especially the case for pro-IAPPβ, with an AUROC of 0.89, which is in the range of accuracy for the most advanced immune assays for markers considered central to AD pathogenesis, such as Aβ (0.78) [64], soluble Aβ oligomers (0.89) [65], pTau217 (0.98), pTau181 (0.97) [66], N-terminal Tau fragment (0.95) [67], total Tau (0.78), and neurofilament light (0.87) [68] or even neuronal-derived extracellular vesicles (0.89) [69], which have been proposed as predictors of future AD diagnosis [70]. Given the limited number of plasma samples (19 controls and 10 individuals with AD) examined, the diagnostic performance of the hIAPPβ and hIAPPγ SRM assays should be further validated in larger studies for clinical relevance since IAPP_37_ is known to enter the brain and may be involved in seeding of Aβ amyloid.

## 5. Conclusions

We uncovered hominid-specific peptides derived from IAPP isoforms that potentially could be developed as blood-based biomarkers for early AD and have use as peptide-based anti-amyloid drugs.

## Data Availability

The IAPP isoform cDNA clones for hIAPP-a and hIAPP-g, tryptic peptides, and TaqMan probes are available upon request.

## Supplementary Materials

The following supporting information can be downloaded at: https://www.mdpi.com/article/10.3390/biom13010167/s1, Table S1 (TaqMan probe and primer sequences of human IAPP isoforms) and S2 (Unlabeled and stable isotope labeled IAPPβ and IAPPγ isoform tryptic peptide sequences); Figure S1 (IAPP exon ortholog nucleotide sequence alignments), Figure S2 (hIAPP isoform mRNA levels in islets and testis; and pro-IAPPβ and pro-IAPPγ peptide levels in plasma samples, and Figure S3 (Statistical analysis of hIAPP isoform peptide levels in human plasma samples).
