# Supplementary material for: Novel Hominid-Specific IAPP Isoforms: Potential Biomarkers of Early Alzheimer’s Disease and Inhibitors of Amyloid Formation
Source: Biomolecules. 2023 Jan 13;13(1):167. doi: 10.3390/biom13010167 (PMC9856209; doi:10.3390/biom13010167)
Supplement: Supplementary file 1 [file biomolecules-13-00167-s001.zip › biomolecules-2074943-supplementary.pdf]

Supplementary Information:

Table S1. TaqMan probe and primer sequences of human *IAPP* isoforms.

Isoforms	TaqMan probe	Forward primer	Reverse primer
hIAPP-ex1	AATATCTACATCTACATTTCCAG	CTTTGCACATCTCCAGGTCAGA	TGCCCATTGCTTCTCAAATTT
hIAPPα	CAATGTCAGCAATATCAG	CCCATTGCTTCTCAAATTTTCTTTTA	TCTTTTCTTGAAGCTTTCTTTCTATCAG
hIAPP-ex3a	TTAAAAGTGCTGGATTATTC	GCATTTGCTGATATTGCTGACATT	CCCATTGCTTCTCAAATTTTCTG
hIAPPβ	TGAACCATCTGAAAGCT	TCTCATTGTGCTCTCTGTTGCA	TGGTCCAAACATCTTTCAATGG
hIAPPγ	AAAGAAAATTTGAGAAGCAATGG	GCTGATATTGCTGACATTGAAACAT	GCACAATGAGAAATACTTGCAGCTT

Table S2. Unlabeled and stable isotope-labeled (marked by asterisk) IAPP-β and -γ isoform tryptic peptide sequences and their molecular weights (MW; dal, Dalton) for SRM-MS assay. CAM in the peptide sequences represents carbamidomethylation of cysteine residues to block its oxidation, and (^) the stable isotope labeled amino acid residues (nitrogen-15 for K and R residues).

Peptide name	Peptide sequences	MW (dal)	AA
pro-IAPPβ	H_2N-C[CAM]LDQIPIFTVFQENHQVEK -OH	2345.14	19
pro-IAPPγ	H_2N-QEWIIPVLSR-OH	1240.70	9
fs-IAPPγ	H_2N-SGNATLPHVQR-OH	1179.62	11
pro-IAPPβ*	H_2N-C[CAM] LDQIPIFTVFQENHQVEK^-OH	2353.14	19
pro-IAPPγ*	H_2N- QEWIIPVLSR^-OH	1250.70	9
fs-IAPPγ*	H_2N-SGNATLPHVQR^-OH	1189.62	11

Figure S1: IAPP exon ortholog nucleotide sequence alignments. Red letters and dash lines represent nucleotide substitutions and deletions in different species. Exon splicing acceptor site (AG) and donner site (GT) are bolded and underlined. The exon sizes including splicing sites (AG------GT) are marked by numbers at the right.

Figure S1A

Hominid *IAPP* Exon-4 ortholog alignment

```
Human     AGACAGGAATGGATAATTCCAGTTTTGTCAAGAAATATACTCTTGGAACTTAGAGGGGCAAAGCCAGAACATGAAGCGGGAAAAAAAATCAAAAGGT 96
Chimp     AGACAGGAATGTATAATTCCAGTTTTGTCAAGAAATATACTCTTGGAACTTAGAGGGGCAAAGCCAGAACATGAAGCAGGAAAAAAAATCAAAAGGT 96
Gorilla   AGACAGGAATGGATAATTCCAGTTTTGTCAAGAAATATACTCTGGGAACTTAGAGGGGCAAAGCCAGAACATGAAGCAGGAAAAAAAATCAAAAGGT 96
Orangutan AGACAAGAATGGATAACTCCAGTTTTGTCAAGAAATATACTCTTGGAACTTAGAGGGGCAAAGCCAGAACATGAAGCAGGAAAAAAAATCAAAAAGT 96
Gibbon    AGACAGGAATGGATAATTCCAGTTTTGTCAAGAAATATGCCCTTGCAACTTAGAGGGGCAAAGCCAGAACGTGAAGCAGGAAAAAAATCAAAAA-GT 95
          ***** ***** **** ********************* * ** * ************************ ****** ********   **** **
```

Figure S1B

Primate *IAPP* Exon-4 ortholog alignment

```
Macaque  AGTTACATATTTAGTTTCCAGCATATTTTCAAAAGAAATATGG--TTGGAACTTAGAGGGGCAAAGCCAGAACATGAAGCAGAAAAAAAAATCAAAAG---GT 97
Sabaeus  AGTTACATATTTAGTTTCCAGCATATTTCCAAGAGAAATATGGTCTTGGAACTTAGAGGGGCAAAGCCAGAACATGAAGCAGAAAAAAAAATCAAAAG---GT 99
Baboon   AGTTACATATTTAGTTTCCAGCATATTTCCAAGAGAAATATGGTCTTGGAACTTAGAGGGGCAAAGCCAGAACATGAAGCAGAAAAAAAA-TCAAAAGATAGT 101
Drill    AGTTACATATTTAGTTTCCAGCATATTTCCAAGAGAAATATGGTCTTGGAACTTAGAGGGGCAAAGCCAGAACATGAAGCAGAAAAAAAA-TCAAAAGATAGT 101
Gelada   AGTTACATATTTAGTTTCCAGCATATTTCCAAGAGAAATATGGTCTTGGAACTTAGAGGGGCAAAGCCAGAACATGAAGCAGAAAAAAAA-TCAAAAGATAGT 101
         *******************************************  ******************************************** *******   **
```

Figure S1C

Hominid and primate *IAPP* Exon-5 ortholog alignment

```
Human     AGATGTTTGGACCAAATTCCAATTTTTACTGTTTTTCAAGAAAAGT 46
Chimp     AGATGTTTGGACCAAATTCCAATTTTTACTGTTTTTCAAGAAAAGT 46
Gorilla   AGATGTTTGGACCAAATTCCAATTTTTACTGTTTTTCAAGAAAAGT 46
Orangutan AGATGTTTGTACCAAATTCCAATTTTTACTGTTTTTCAAGAAGAGT 46
Gibbon    AGATGTTTGGGCCAAATTCCCATTTTTACTGTTTTTCAAGAAAAGT 46
Sabaeus   AGATGTTTGGACCAAATTCCAATTTTTAT-GTTTTTCAAGAAAAGT 45
Lemur     AGATGTTTGGGTCAAATTCTAATTCTTGCTGGTTTTCAGGAAAAGT 46
Marmoset  AGGTATTTGGACCAAATTCCAGCTTTTCCTGATTTTCAAGAAAAGT 46
```

Figure S1D

Hominid *IAPP* Exon-1 ortholog alignment

```
Human   AGCTTTGCACATCTCCAGGTCAGACCTATCCTAATATCTACATCTACATTTCCAGGT 57
Chimp   AGCTTTGCACATCTCCAGGTCAGACCTATCCTAATATCTACATTTCCA------GGT 51
Gorilla AGCTTTGCACATCCCCAGGTCAGATCTATCCTAATATCTACATCTACATTTCCAGGT 57
        ************* ********** ****************** * **
```

Figure S2: (A) hIAPP isoform mRNA levels in islets and testis and (B) pro-IAPPβ and pro-IAPPγ peptide levels in plasma samples (n=28).

Figure S2A

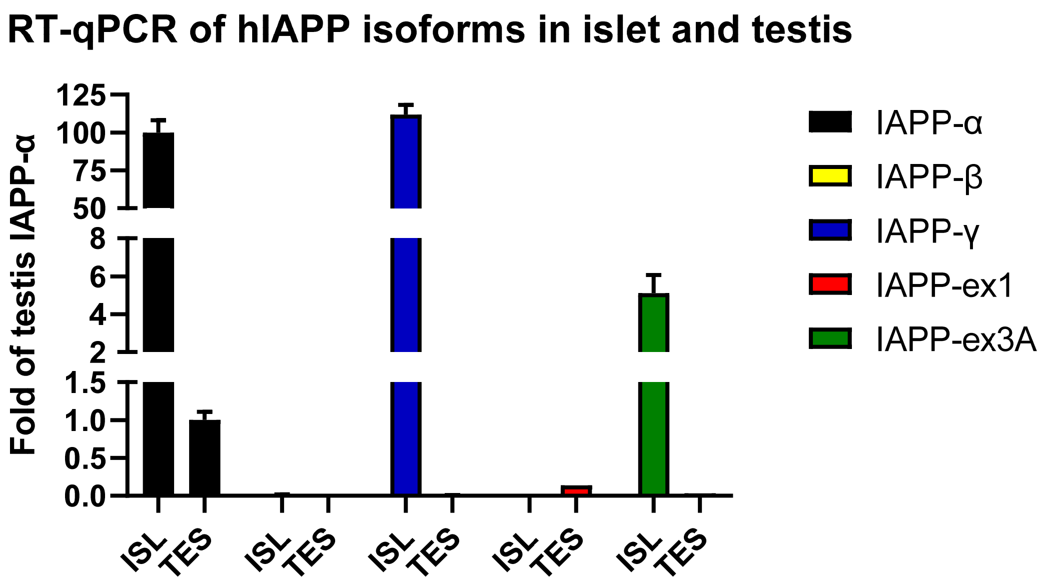

Figure S2B

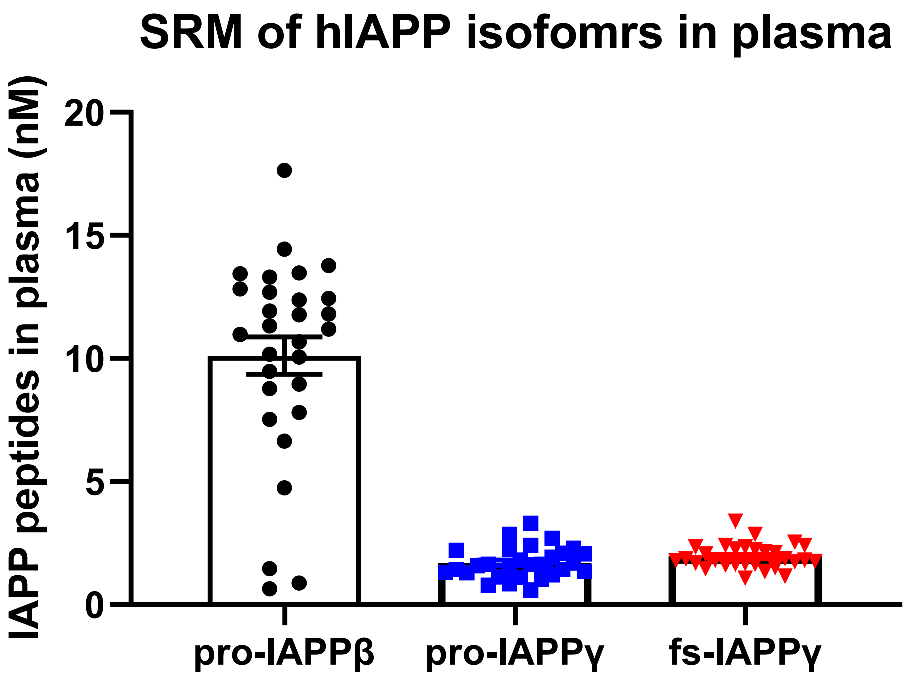
SRM of hIAPP isofomrs in plasma
IAPP peptides in plasma (nM)
20
15
10
5
0
pro-IAPPβ
pro-IAPPγ
fs-IAPPγ

Figure S3: Statistical analysis of hIAPP isoform peptide levels in human plasma samples. (A) Correlations of IAPP-ELISA with SRM peptides in plasma samples. (B) Partial least squares-discriminant analysis (PLS-DA) of pro-IAPPβ, pro-IAPPγ, and fs-IAPPγ in plasma samples of the control and AD. The first two components of the hIAPP isoforms explain 99% of the total variance and (C) they clearly separate plasma samples of control and AD.

Figure S3A

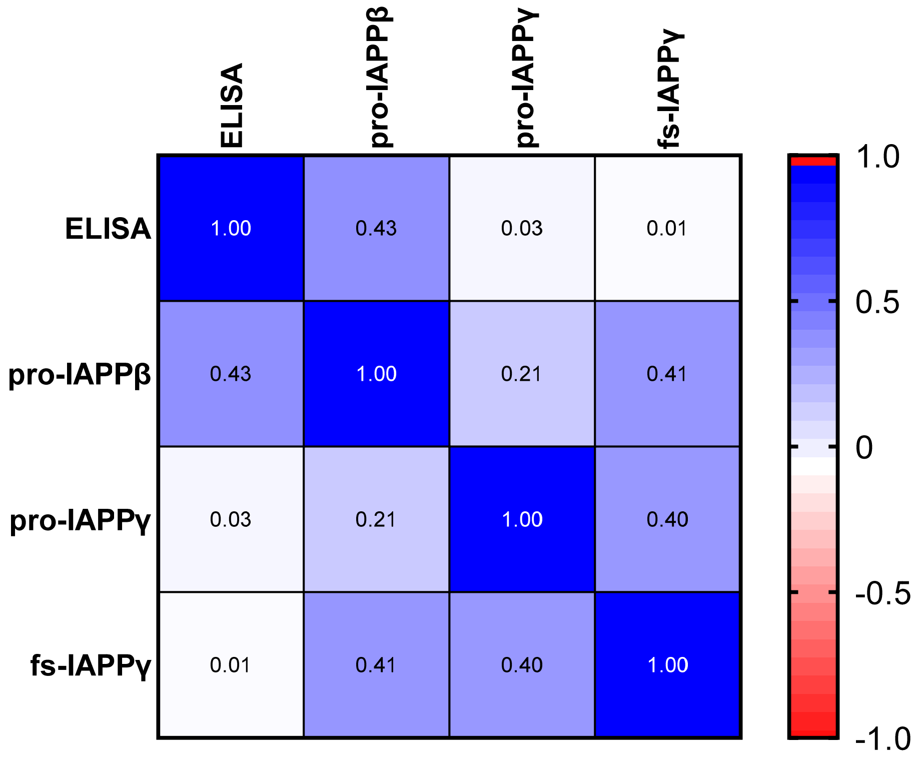

Figure S3B

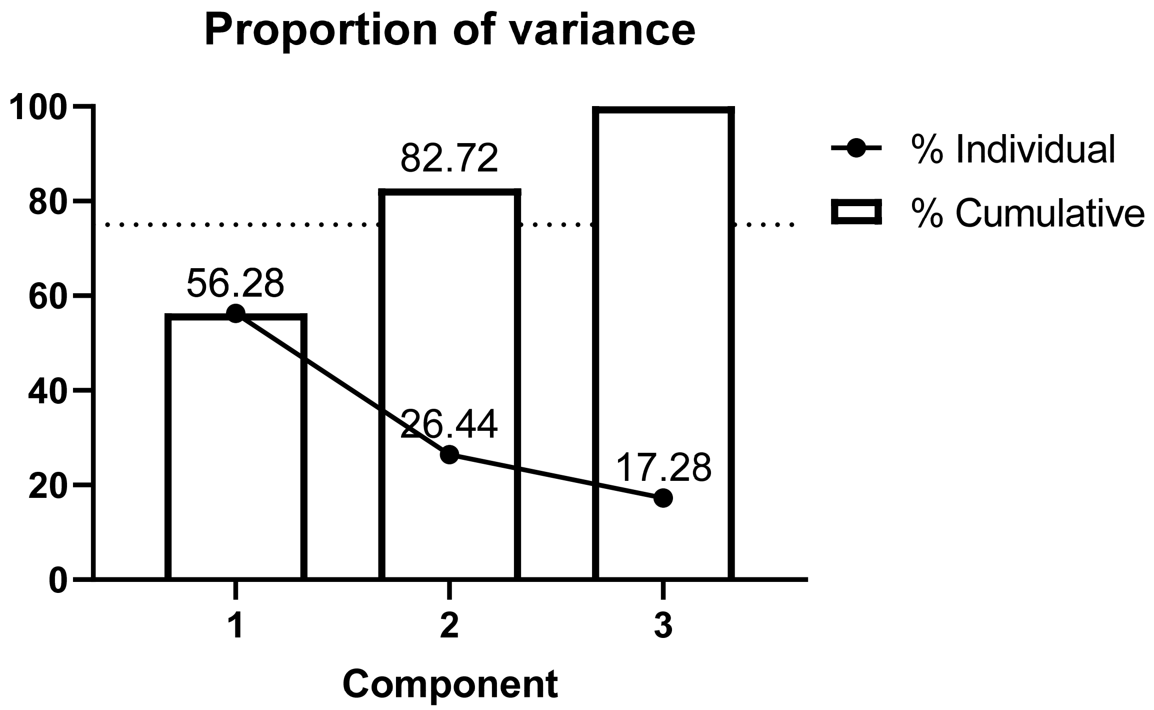
Proportion of variance
100
80
60
40
20
0
82.72
56.28
26.44
17.28
1
2
3
Component
% Individual
% Cumulative

Figure S3C

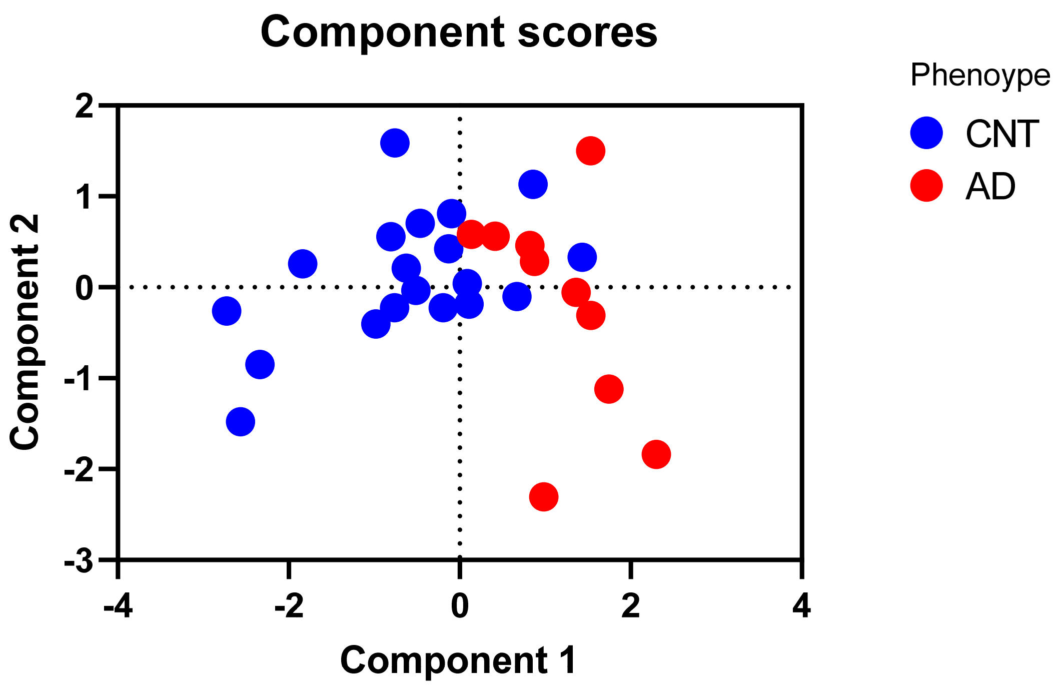
Component scores
Component 2
Component 1
2
1
0
-1
-2
-3
-4
-2
0
2
4
Phenoype
CNT
AD
